# BioLegato: a programmable, object-oriented graphic user interface

**DOI:** 10.1186/s12859-023-05436-4

**Published:** 2023-08-21

**Authors:** Graham Alvare, Abiel Roche-Lima, Brian Fristensky

**Affiliations:** 1 Access Norwest Co-op Community Health, Winnipeg, Canada; 2 RCMI Program, Medical Science Campus, University of Puerto Rico, San Juan, Puerto Rico, USA; 3 Department of Plant Science, University of Manitoba, Winnipeg, Canada

**Keywords:** Graphic user interface, User experience, Data pipelining, Sequencing, Genomics, Transcriptomics

## Abstract

**Background:**

Biologists are faced with an ever-changing array of complex software tools with steep learning curves, often run on High Performance Computing platforms. To resolve the tradeoff between analytical sophistication and usability, we have designed BioLegato, a programmable graphical user interface (GUI) for running external programs.

**Results:**

BioLegato can run any program or pipeline that can be launched as a command. BioLegato reads specifications for each tool from files written in PCD, a simple language for specifying GUI components that set parameters for calling external programs. Thus, adding new tools to BioLegato can be done without changing the BioLegato Java code itself. The process is as simple as copying an existing PCD file and modifying it for the new program, which is more like filling in a form than writing code. PCD thus facilitates rapid development of new applications using existing programs as building blocks, and getting them to work together seamlessly.

**Conclusion:**

BioLegato applies Object-Oriented concepts to the user experience by organizing applications based on discrete data types and the methods relevant to that data. PCD makes it easier for BioLegato applications to evolve with the succession of analytical tools for bioinformatics. BioLegato is applicable not only in biology, but in almost any field in which disparate software tools need to work as an integrated system.

## Background

While there exists a wealth of freely-available bioinformatics tools, their number and diversity present several challenges to biologists. By analogy to enzymes, each tool has several rate-limiting steps: installation, reading the documentation, experimenting with the commands needed to run the tool, and formatting of input files. Further complications arise if the plan is to use output from one program as input for the next. In that case, a new learning curve is needed for the next tool.

One solution is to build applications that put together different steps, often using a combination of internal functions and calls to external programs. For example Ugene integrates a combination of intrinsic functions, calls to programs installed with Ugene, and calls to web services [1]. Writing applications of this type require mastery of the biology, sophistication in algorithmic aspects of the work, and in construction of intuitive graphical user interfaces (GUI).

Alternatively a client-server approach implements the GUI as a web-based client with a server-based back-end to carry out analytical functions. For example, the tools at EMBL-EBI use Javascript and HTML to create simple web front ends to an array of programs [2]. More elaborate is the NCBI site which integrates the Genome Data Viewer [3] with tools such as BLAST [4] and the Entrez databases [5]. Another example is Galaxy which allows a user to upload datafiles which are processed using workflows controlled through a web interface [6]. Web menus for tools are built using an XML format for specifying parameters, inputs, outputs, help pages etc.

However, web interfaces are at best a poor compromise when it comes to usability. A web page typically takes up most or all of the screen, making more difficult side by side comparison of different types of information such as manuscripts, data files, results, or spreadsheets. Larger screens, and even dual monitors, have become popular in data science, so the “one window owns the screen” model negates the benefits of these devices. Web interfaces also suffer from the fact that they cannot directly access local disk files (albeit for obvious security reasons). This means that if a researcher uses different web applications at a variety of sites, the workflow will be punctuated by an upload, run, download cycle at each step. This becomes especially problematic with large datasets for which file transfer times are significant. Finally, the user can’t add new functions to a web-based GUI.

The Genetic Data Environment (GDE) [7] resolved the tradeoffs between ease of software coding and having an intuitive and versatile GUI. GDE was a programmable GUI whose sole function was to execute commands. Rather than hard-coding each task into GDE, specifications for creating parameter menus would be read at runtime from files which used a simple syntax to define GUI elements like buttons, sliders choosers and labels. However, GDE is no longer supported, worked only with sequence data, and had limited functions for specifying GUI components using a now obsolete C toolkit. Nonetheless, the core ideas behind GDE are still sound and are the starting point for the current work.

We have previously described PCD [8], a simple language for specifying menu components such as choice buttons, file choosers, option choosers, numerical settings etc. PCD is defined in a formal grammar, implemented using javacc to generate Java code prior to compilation [8]. Using PCD, we now demonstrate the creation of a family of BioLegato applications (Table 1), each specialized for different types of data, such as DNA or protein sequences or alignments, phylogenetic trees, or sequencing read files. These BioLegato applications have replaced GDE as the GUI layer of our BIRCH bioinformatics system [9]. Organization of these applications by Object-Oriented principles makes BioLegato applications more intuitive to use. By eliminating most of the learning curve for each program, BioLegato makes it easy for the biologist to experiment with different programs and methods at each stage of the analysis. In many cases, output from one step pops up in a new BioLegato instance, making it easy to go from one step to the next.

**Table 1:** .

Application	Data type	Method examples
birch	NA	Launches other BioLegato applications
birchadmin	NA	BIRCH administration tasks
bldna	Nucleic acid sequences	Primer design; restriction search; Pairwise similarity; multiple alignment
blprotein	Protein sequences	Aa comp; pairwise similarity; Multiple alignment
blnalign	Aligned nucleotide sequences	Sequence logos; phylogeny
blpalign	Aligned amino acid sequences	Sequence logos; phylogeny
blnfetch	Nucleotide sequence metadata	Retrieves nucleic acid sequences from NCBI
blpfetch	Protein sequence metadata	Retrieves protein sequences from NCBI
blncbi	Query terms	Returns lists of sequences from NCBI
bltree	Phylogenetic trees	consensus trees; draw trees
blmarker	Molecular marker data	Phylogeny
blreads	Sequencing read files	Preprocessing of reads and assembly of genomes and transcriptomes
bltable	Generic tabular data	Basic spreadsheet operations
blgeneric	NA	Demonstration of BioLegato as an abstract class

## Implementation

The current BioLegato code requires Java 1.8 or higher. The code for BioLegato’s PCD parser is maintained as a formal grammar in Javacc, which generates the Java code for the parser as part of the compilation [8]. Thus, additions to the parser are implemented as changes to the grammar.

As part of the BIRCH system, BioLegato has been tested on low end computers running Linux or MacOS. On High Performance Computing (HPC) systems, we routinely use BioLegato on the Univ. of Manitoba Red Hat Enterprise Linux system [10]. Users run full Xfce desktop sessions on multiuser login hosts through the Thinlinc client (Cendio AB Linkoping, Sweden). For resource-intensive jobs, the user logs into one of 15 compute nodes (256 Gb RAM, 64 cores) using ssh -X, and launches BioLegato from the command line. BioLegato displays on the user’s desktop session, but all computation is done on the HPC node. Because all user directories are mounted by NFS both to login hosts and compute nodes, files do not need to be transferred between hosts.

## Results

### BioLegato work cycle

**Fig. 1:**
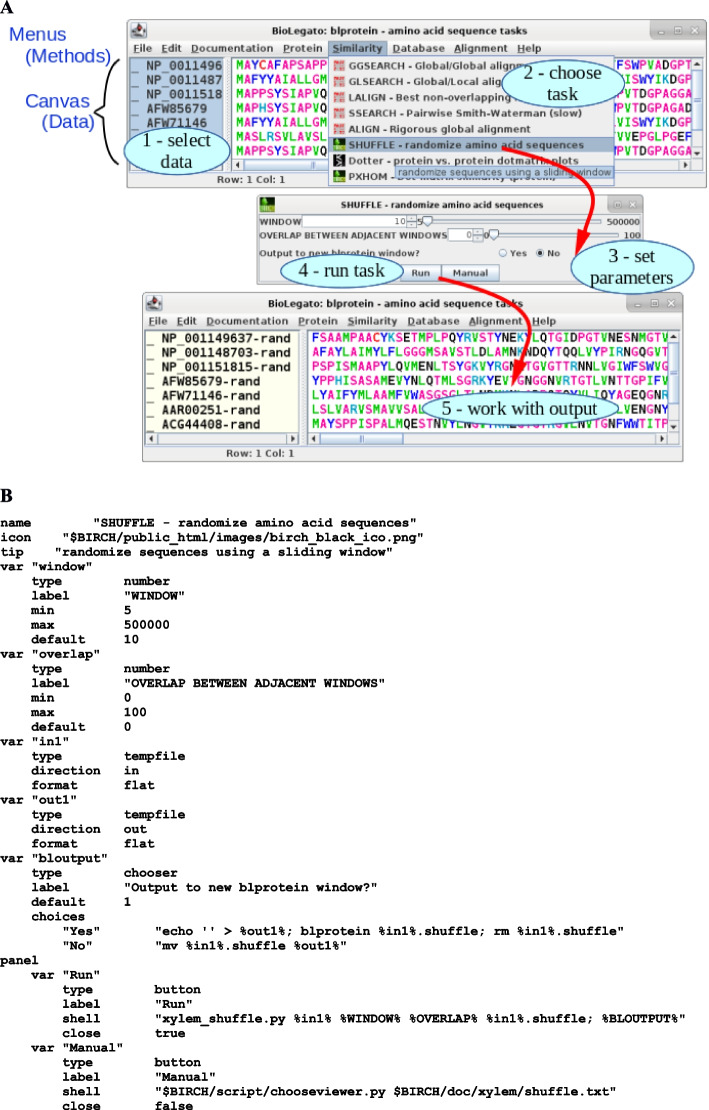
**A** The Biolegato work cycle. (Top) User selects a sequence and chooses to run Shuffle from the Similarity menu. (Middle) The Shuffle menu lets the user set two numerical parameters and a Yes/No parameter. After clicking on ‘Run’, the selected sequence is exported to a file, used as input for Shuffle. Because the user answered ‘Yes’ to “Output to new blprotein window?“, the output file is loaded into a new instance of blprotein, which pops up on the screen (Bottom). **B** PCD code which implements the Shuffle menu. The first 3 lines specify a title for the menu window, an icon to identify the package from which the program is derived, and a tool tip. Note that “$BIRCH” refers to an environment variable listing the path to the BIRCH directory, in which the BioLegato directories are found. This illustrates the point that any part of a PCD menu item may contain references to environment variables to be substituted into the menu. Each “var” item declares a parameter to be used for calling the program. For example, “window” refers to the size of the sliding window for randomization. “in1” and “out1” respectively are the names of temporary files to be used for input and output of by xylem_shuffle.py. In the “panel” section, the “Run” button will execute the command found on the shell line. This is a command template, into which the values of the variables will be substituted. Thus %in1% will be substituted with an automatically generated name for a temporary input file, and %WINDOW% will be substituted with the number set in the WINDOW parameter. %BLOUTPUT% is a placeholder which will be substituted with the command fragment specified in the “bloutput” variable. In the example, the user chose “Yes”, so the shell command will be substituted with the command fragment from “bloutput”, which opens the output file in a new instance of blprotein. If “No” had been chosen, the temporary output file would be renamed to %out1%, causing the output to be read into the current BioLegato object. If the var definition for “out1” had included “overwrite true”, the entire contents of the canvas would be replaced by the contents of the out1 temporary file. These features make conditional execution of code possible, allowing for more complex behavior

Use of BioLegato is five step workcycle: select data, choose a task, set parameters, run the task, and work with the results (Fig. 1A). Output could go to a viewer such as a Web browser, PDF viewer, or text editor, to a 3rd party program such as a multiple alignment editor, or to another BioLegato instance. The latter is one of the most powerful aspects of BioLegato: the ability to keep the output machine readable, so that the user has many choices for the direction of downstream analysis. In practice, a series of tasks might result from running a series of BioLegato work cycles, a process we refer to as ad hoc pipelining. Ad hoc pipelining is distinct from and complementary to preprogrammed workflows. While workflows exist to automate processes that are the same every time, ad hoc pipelining is an exploration in which the user can experiment with different approaches to learn from the data, instructing the direction of downstream analysis. The name “BioLegato” is an analogy to legato passages in music, in which a theme flows smoothly from one note to the next.

### BioLegato facilitates rapid development of new applications based on object-oriented concepts

The concept of objects is foundational to software engineering [11], as exemplified in Object-Oriented (OO) languages such as Java and C++. The goal of the OO philosophy is to create data objects which model things in the real world by packaging together all data associated with those things. More formally, a class is a template for creating objects. Each class specifies the types of data contained in all objects of that class, along with the methods or functions specific for objects of that class. Whereas a class is an abstract concept, any number of objects may be created as instances of that class.

We have designed BioLegato to bring the disciplines of OOP to the user experience, for most of the same reasons that programmers organize their code into objects. Objects create a coherent model of biological entities, as close as possible to how the biologist thinks about them. Objects are therefore intuitive to work with. As well, ongoing development of BioLegato objects is easy because objects are easy to extend. At this writing our BIRCH system [9] includes 14 applications implemented using BioLegato (Table 1).

**Fig. 2:**
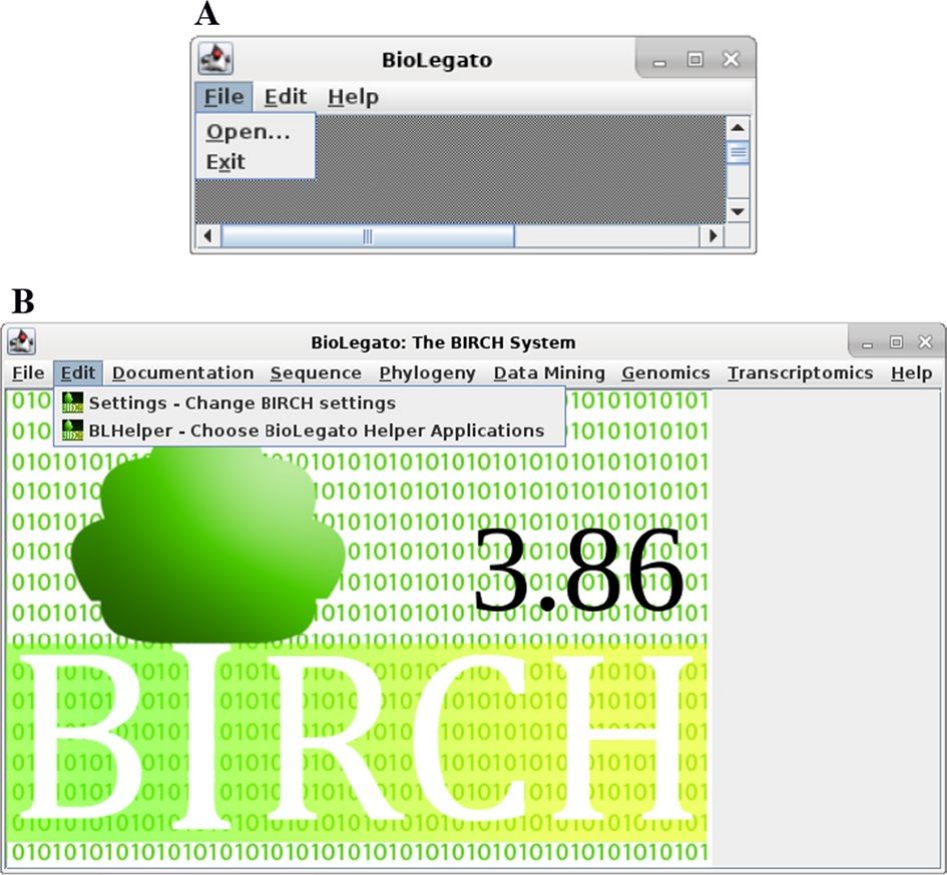
**A** blgeneric **B** birch launcher

As shown in Fig. 1A, BioLegato displays data in the canvas, and organizes methods in PCD-coded menus. Typically, BioLegato is run through a shell script that specifies one or more directories from which to read the PCD menus, and specifies a canvas, implemented as a Java plugin. Currently there are 3 canvases: the image canvas, which displays a simple bitmap image, the sequence canvas, which allows the user to edit sequences and multiple alignments, and the table canvas, which displays data in a simple spreadsheet. To demonstrate that all functionality is loaded at runtime, blgeneric (Fig. 2A) is a minimal BioLegato application that loads a dummy background image and no PCD menus. Figure 2B shows birch, which launches BioLegato and other GUI applications for the BIRCH system, illustrating use of the image canvas.

In blprotein (Fig. 1A), the data are amino acid sequences displayed in the sequence canvas. The methods are the different tasks that can be run when one or more sequences are selected. These include tasks such as doing a hydropathy plot, secondary structure prediction, or amino acid composition. They would not include, for example, a restriction site search, which makes no sense in the context of proteins. By the same token, bldna is a BioLegato application for DNA or RNA sequences. Bldna has tasks such as primer design or a restriction site search. Even where the same program can utilize either protein or DNA, the BioLegato menus can be customized for bldna or blprotein. For example, SSEARCH, which does rigorous Smith-Waterman sequence alignments, would give the user a wide choice of protein scoring matrices if run from blprotein, and DNA scoring matrices if run from bldna.

Separating DNA and protein tasks into different applications makes sense to the biologist. Programs such as BLASTP or TBLASTN, in which the query is an amino acid sequence are run from blprotein, whereas BLASTN or BLASTX or TBLASTX, in which the query is DNA, are run from bldna.

### Menu components for BioLegato are read from files at runtime

The core concept of BioLegato is to build a command for running an external program by substituting options set in a GUI menu into a template command. The blprotein application will be used as an example. blprotein is launched by a shell script that tells the locations of all data files specifying the menus and canvas to be used (BL_DATA_DIR) as well as the location of the Java BioLegato executable (BIOLEGATO_HOME). In the example, blprotein is part of the BIRCH system, so these two variables refer to folders within BIRCH, using the $BIRCH environment variable. For implementation of a BioLegato application outside of BIRCH, $BIRCH could be changed to specify a different folder. The last line launches the application.
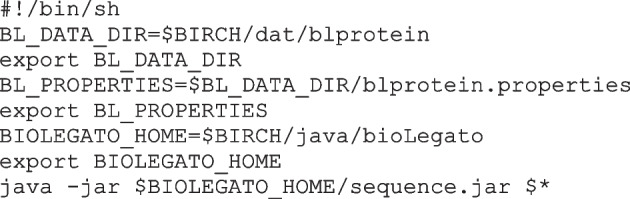


The blprotein.properties file contains the line
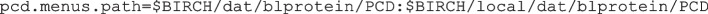


which tells BioLegato the location of folders (directories) containing PCD menu files.

Dropdown menus are created simply by making a series of folders and subfolders within the PCD folder. BioLegato will use the name of each folder as the name for each dropdown menu (e.g. Alignment, Database etc.). The contents of the PCD folder/sub-folder structure for blprotein is given below. For brevity, we don’t show the contents of all subfolders for each dropdown menu. Only Similarity subfolder (indented) is shown.
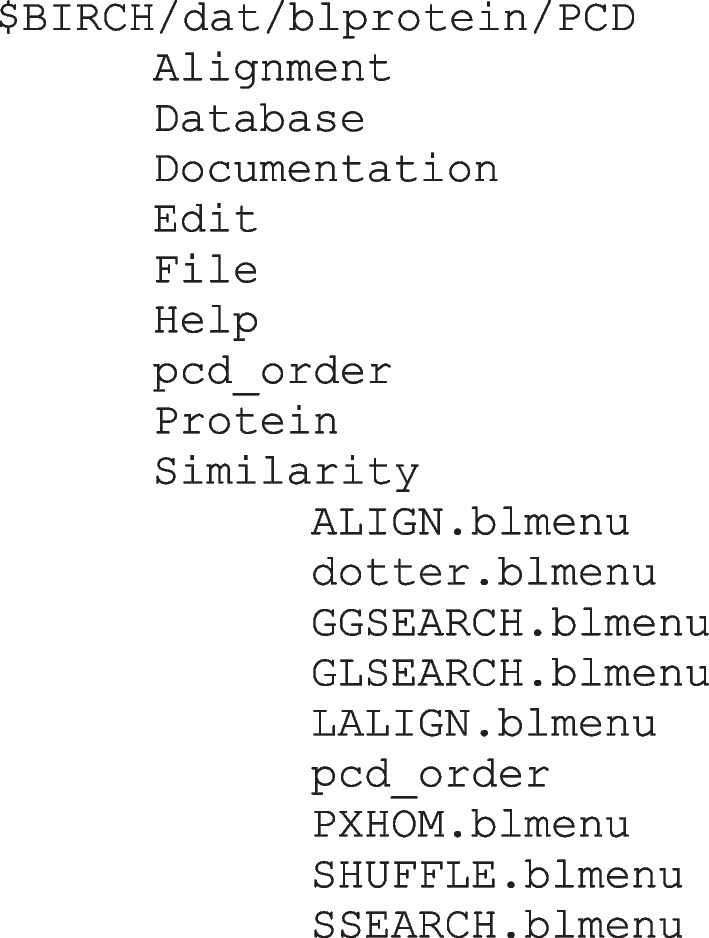


The pcd_order file in the PCD folder specifies the left to right order of the dropdown menus by listing each menu in order (Fig. 1A):
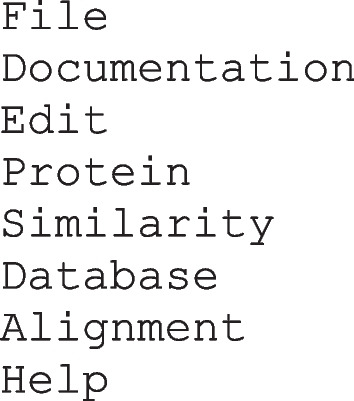


In turn, each subfolder contains one or more .blmenu files, and a pcd_order file to tell the order, top to bottom, in which the programs appear in the dropdown menu e.g. GGSEARCH, GLSEARCH, ALIGN etc. (Fig. 1A).

Although PCD is a rigorously defined language in the formal sense [8], creating PCD menu for running a new program is more like filling out a form than writing code. Typically, one would create a new blmenu file by copying an existing one and then modifying it to call a new program.

The layout and behavior of each parameter menu for a program is specified in a .blmenu file. Figure 1B gives the PCD code for running xylem_shuffle. The top three lines specify the name, or title for the menu, the file path for the small icon to include in the menu, and the text of a tool-tip (blue box in Fig. 1A.) The rest of the .blmenu file works toward building a command line to be executed when the Run button is clicked. Each “var” component specifies the type of widget to appear in the menu.

To make PCD more understandable, PCD borrows from Python, requiring indentation of lines that are part of a menu component, such as var or panel. For example, “window” lets the user set the size of a sliding window to be used for local randomization of amino acids. In the shell command, the value of each var item is substituted into the command where the name of the var is enclosed in percent (%) symbols. Thus, %WINDOW% on the shell line will be substituted with the value set by the user in “window” part of the menu.

Similarly, the “bloutput” variable contains two choices for code to be substituted into the command, for either writing output to a file, or displaying it in an application. In summary, BioLegato creates the command that you would have typed at the command line, but eliminates the need to carefully study the command syntax, as well as eliminating typing errors.

### PCD allows flexibility in GUI design and output visualization

**Fig. 3:**
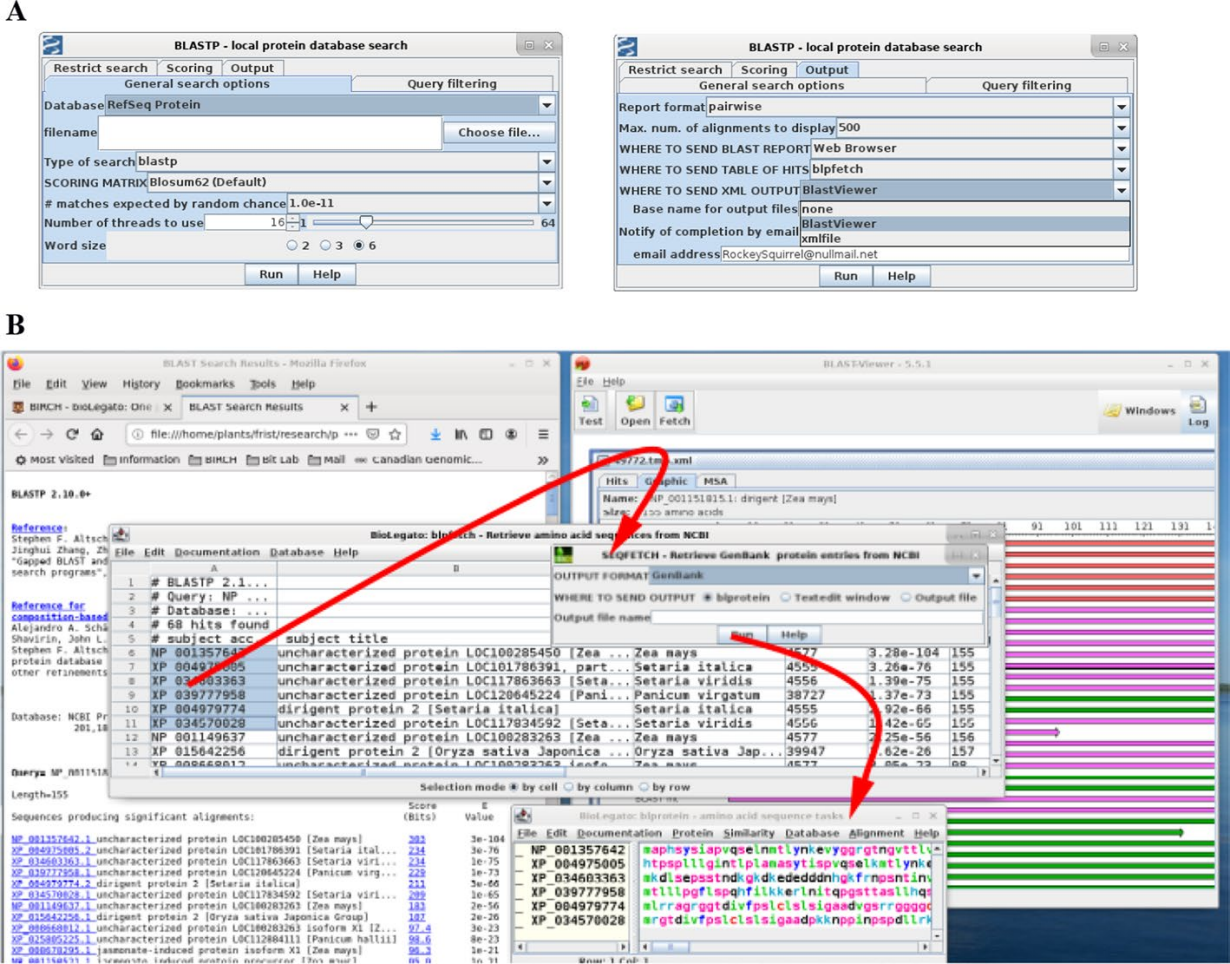
BLAST workflow. **A** Examples of tabbed-panes to organize large numbers of parameters. **B** Output pops up in web browser (left), blpfetch (center) and BlastViewer (right). In blpfetch, user can select hits to retrieve, and GenBank entries are retrieved to a blprotein object (bottom center)

For programs with many parameters, the PCD tabbed panes make it possible to organize parameters into groups. Figure 3A shows the General search options pane and the Output pane. Note that the Number of threads field in the General search options pane sets the maximum range of the slider to the number of cores on the system. Rather than being hard-coded in PCD, this slider is implemented as follows:
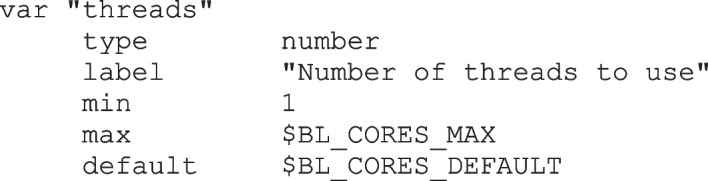

The two environment variables $BL_CORES_MAX and $BL_CORES_DEFAULT are set by the blprotein wrapper script prior to launching BioLegato (not shown). Thus, these two numbers will be correct for each system on which blprotein is run.

In Fig. 3B, BLAST output goes to a web browser, BlastViewer [12], and to blpfetch, a BioLegato application that displays protein search results using the table canvas. The table canvas illustrates some of the usability advantages of desktop applications versus web applications. The NCBI Web BLAST implements tabular output using HTML and Javascript. In blpfetch, one can rapidly select BLAST hits either by dragging or a combination of drag, SHIFT, scroll and click, to rapidly select hundreds or even thousands of hits made contiguous from sorting, cutting and pasting. The NCBI web form only supports selecting sequences one by one, or selecting all. While it is true that the table could be exported to a CSV file and then opened in a spreadsheet, the final set of accession numbers would still have to be saved to a file and read in using Batch Entrez, to accomplish the same result. Additionally, browser-based applications have the intrinsic problem that there is usually a substantial wait for the page to reload any time a change is made. In desktop applications like blpfetch, the user has more of a sense of working directly with the data because operations such as sorting happen almost instantly.

### The table canvas can represent a diversity of data types

**Fig. 4:**
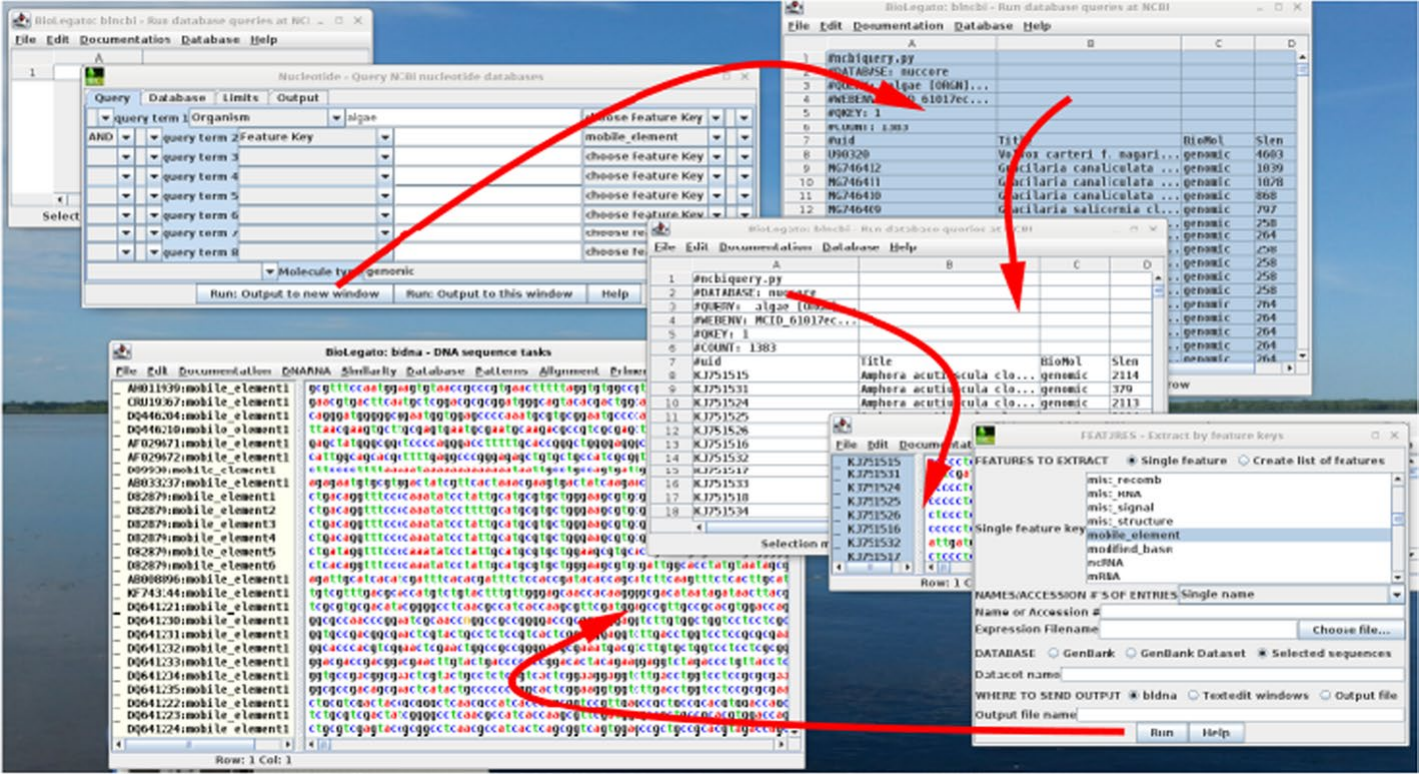
Workflow for creating a database of mobile elements from algae. Clockwise from top left: Keyword search is done in blncbi query builder. In the nucleotide search field, the user chooses Organism and types in “algae”. Since automated extraction of features is desired, the boolean operator AND is chosen, and the second search field set to “Feature Key mobile_element”. Thus, only GenBank entries with the mobile_element features key would be found. For more complex queries, left and right parentheses could be chosen to group terms together. Since genomic sequences are desired, the Molecule field is set to genomic. When the search is launched, the Entrez search expression “algae [ORGN] AND mobile_element [FKEY] AND biomol genomic [PROP] AND 1:500000[SLEN]” is sent to NCBI using ncbiquery.py, a Python script implementing the NCBI Eutils API. The Entrez document summary for 1383 GenBank entries matching the expression is retrieved to a new blncbi object. To better understand the species distribution of mobile elements, the user sorts by species. GenBank entries are retrieved to a bldna object. From the GenBank entries, FEATURES extracts 37,484 mobile_element sequences to a new bldna object

 The versatility of using PCD to create complex GUIs is illustrated in Fig. 4 in which the user creates a database of transposable elements from Rhodophyte algae in a few clicks. The workflow begins by building a search expression using the blncbi query builder, in which search terms can be combined using boolean operators such as AND, OR and NOT, and grouped using parentheses. The spreadsheet capabilities of the table canvas make it easy to browse through even thousands of lines of output. The comparable workflow at the NCBI web site would have resulted in 70 pages of output. In BioLegato, hits can be selected with the mouse and retrieved in a single step to bldna, and the mobile_element sequences extracted to a new bldna object for downstream analysis.

**Fig. 5:**
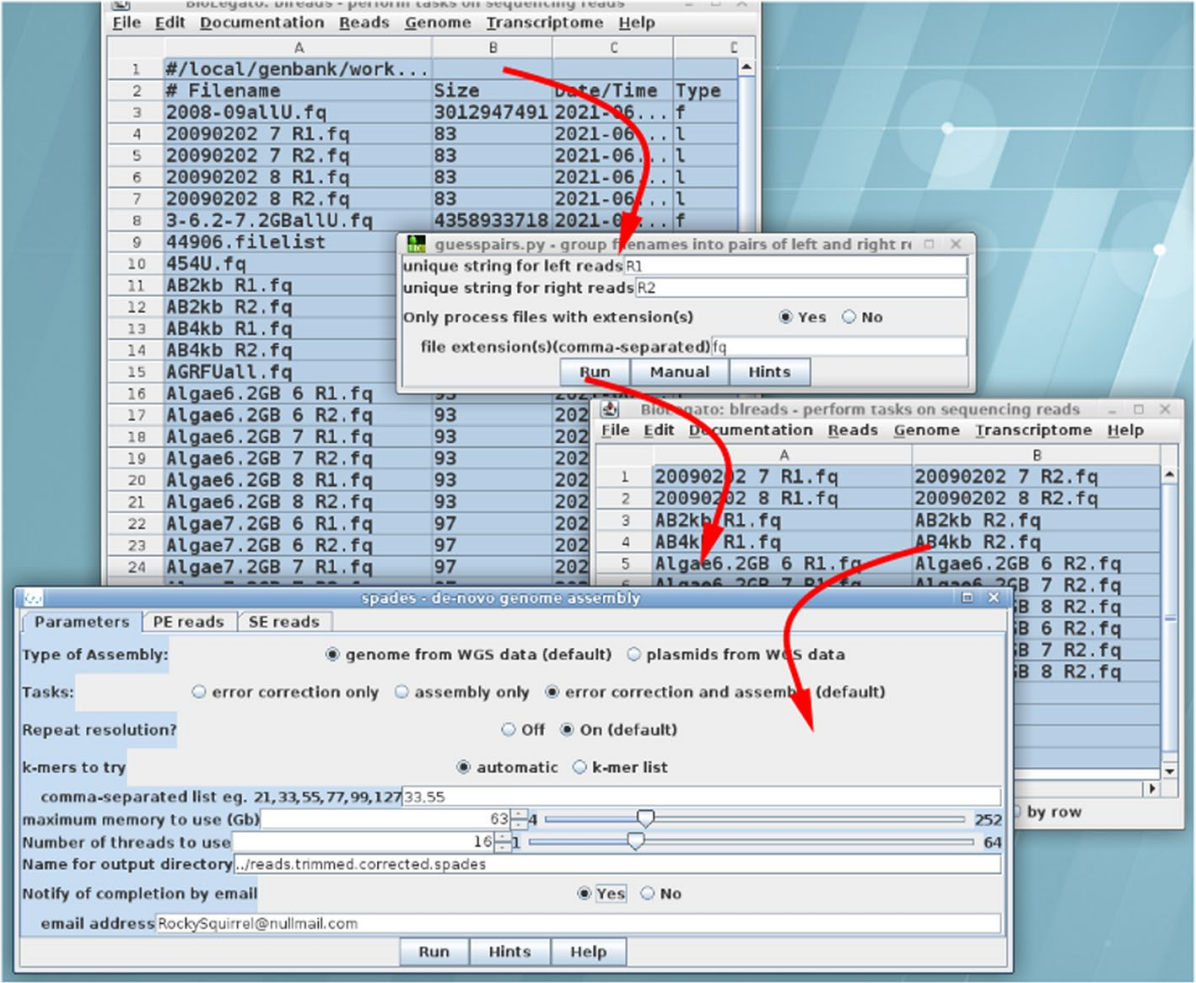
Excerpt from a genome assembly workflow. Clockwise from top: All files in the current directory are selected. Guesspairs.py distinguishes forward read files from reverse read files based on the unique strings R1 and R2. Only fastq files with the “fq” file extension will appear in output. The output from guesspairs.py is a new blreads object with forward and reverse reads paired in two columns. Where only 1 read file is present, it is treated as a single read file. After setting parameters in the Spades menu, the Run button launches a Python script which constructs the command line to run the Spades pipeline using the selected files

 Object-oriented design strives to make objects look as much like the real-world thing as possible. Since high throughput sequencing works with read files, blreads was built to resemble a file manager. Indeed, early in development we concluded that in addition to functions for processing reads and assembling genomes and transcriptomes, blreads should also have typical file functions such as compress/uncompress, delete, rename, open directory, or view file. Figure 5 demonstrates that the file manager format lends itself to an intuitive way of grouping files for forward and reverse reads. The user runs guesspairs.py to generate a new blreads object with forward and reverse read files in two columns. In the example, when the user launches Spades [13], BioLegato saves the file list as a tab-separated value (TSV) file. A custom script reads the file and generates the appropriate command to group forward and reverse files on the spades command line. In practice, we have found that programs that work with read files have a diverse array of ways to specify read pairs. For example, spades would specify forward and reverse files on the command line as “-1 seqs-R1.fq -2 seqs-R2.fq”, while SOAPdenovo2/Megahit [14] requires the user to create a config file in which read pairs are specified on two lines reading “q1 = seqs-R1.fq” and “q2 = seqs-R2.fq”. By hiding what can be a maddening array of command line syntax specifications in helper scripts, BioLegato saves the user hours of trial and error just to get each program in the pipeline to work.

### Implementation of new BioLegato applications

BioLegato lends itself to developing new applications by copying and modifying existing ones. Each application is launched by a shell script that sets any environment variables needed, including the location of the BioLegato Jar file and the locations of PCD menu files. Thus, a new BioLegato application is created by modifying copies of the shell script and PCD menu files to call any programs on the system.

BioLegato also provides for local customization to add programs or modify how they are run at each site. For example, in BIRCH, the blreads.properties files contains the line “pcd.menus.path=$BIRCH/dat/blreads/PCD:$BIRCH/local/dat/blreads/PCD”. At run-time, BioLegato will load PCD menus from PCD directories in the order shown. If PCD menus file in the “local” directory tree has the same name as one previously read, the local menu file will supersede the one previously read. Thus PCD items added locally to a BIRCH installation are seamlessly integrated into the BioLegato application.

## Discussion

This paper has focused on how PCD makes it far easier to add functions to existing applications, or create new applications, compared to conventional compiled languages or web interfaces. However, even non-programmers can benefit from the family of BioLegato applications simply by installing the BIRCH system, cited in the Availability section. The getbirch install wizard downloads and installs BIRCH within a matter of minutes. BIRCH really shines on multi-user HPC platforms with a centrally-installed copy of BIRCH for all users. As the need arises for new programs, the person managing the local BIRCH system adds a new BioLegato menu to $BIRCH/local, and the new program will appear in BioLegato for all users. In this way, BioLegato makes it easy to tailor the system to the needs of the local user community.

Most GUI software is Object Oriented to some degree, because the ideas behind the OO philosophy reflects on how people think about real world things. For example in Jalview the core data type is a multiple sequence alignment [15]. However, Jalview can also read unaligned sequences and perform a multiple alignment. The OO paradigm breaks down because it is possible to read in a set of unaligned sequences, and still do things like highlighting conserved positions, which is meaningless if sequences are not aligned. The BioLegato applications adhere more deliberately to OO. For example, a multiple protein alignment is run using blprotein, and the aligned output goes to blpalign. Displaying conserved positions can only be done in blpalign, and not in blprotein.

Almost every area of biology has become dependent on complex and sophisticated software tools, and there is an increasing acceptance among biologists of the need for command line tools [16]. This has lead many younger biologists to learn a minimal amount of programming, usually in Python or R. Most genomics tools don’t have a GUI, because writing GUIs requires a great deal of extra work and an additional skill set for programmers. This skill set typically takes years to attain, including the fundamentals of procedural, functional or Object-Oriented programming, extensive language syntax, algorithmic design for analytical components of the program, best programming practices, plus a plethora of development tools such as make, git, and use of an integrated develpment environment (IDE), as well as an application programming interface (API) with tools for GUI development. By the same token, programming for Web interfaces requires similar fundamental knowledge of programming, plus HTML, knowledge of a web scripting language such as PHP or Javascript, as well as knowledge of web servers.

While the learning curve for adding programs to BioLegato is not zero, the expertise required is far less than that required for other approaches to GUIs. Any bioinformatician who supports software for a lab, department, or core facility would find the addition of new programs to a BioLegato application trivial. In such a centrally-managed multiuser environment, all users benefit from local additions to BioLegato. Even without reading the formal syntax documentation, a biologist with minimal scripting experience could quickly learn to add functionality to BioLegato using a copy, modify and test strategy. BioLegato follows the Unix design philosophy, summarized by MD McIlroy, that any tool should “do one thing and do it well” [17]. BioLegato is the logical bridge between the need for sophisticated tools that each do one thing well and usability. In OO parlance, BioLegato “hides the implementation”, giving the user easy access to a wealth of bioinformatics tools in the GUI layer, while the same tools can be run from the command line if desired.

While the idea of separating the GUI and analytical functions as distinct software layers is nothing new, the GUI layer is usually web based. The down side of web interfaces is seen both in the limitations of HTML and Javascript, but also in the latency associated with reloading pages at each step. In BioLegato, the user has more of a feel of working directly with the data.

BioLegato was designed to be completely agnostic of the analytical layer. For example, blncbi follows the client-server model, with BioLegato as the local client, and the server end being the NCBI Entrez system. BioLegato applications could easily be built to utilize any web services for which a remote API exists.

A key strength of BioLegato as an independent GUI layer is that BioLegato provides a seamless way of unifying disparate 3rd party programs from different authors, written in different languages. In the examples, BioLegato applications called programs written in bash, Perl, Python, Java, C, C++, Ruby and Go. BioLegato is robust to failure of the external programs it calls, and improves as new versions of existing programs are updated, or as new programs are added using PCD.

Although PCD is a small language [8], we have demonstrated its ability to generate rich and complex behaviors. BioLegato applications are built by combining PCD menus and a canvas. The birch and birchadmin applications (Table 1) are launchers for other programs, so in this case a simple image canvas suffices. The sequence canvas is used for DNA and protein sequences, in bldna and blprotein, DNA and protein alignments in blnaligh and blpalign (not shown), but also in bltree, where phylogenetic trees in the New Hampshire format are represented as sequences (not shown). Because a great deal of scientific data can be represented in tables, the table canvas is by far the most versatile canvas. It has been used to represent BLAST hits in blnfetch and blpfetch, molecular marker data in blmarker (not shown), and for many diverse file types, as implemented in blreads. Examples of all of these BIRCH applications can be seen at the BIRCH tutorials site at [18].

BioLegato uses OO concepts both at the level of the Java code for BioLegato itself, and in the look and feel of BioLegato applications. Both aspects of BioLegato lend themselves to future development. Because canvases are plugins, two new types of canvas would greatly extend the scope of what BioLegato can do. Because relational databases structure everything in tables, the table canvas might be extended into a database canvas, making BioLegato a client for relational databases. An XML canvas would enable BioLegato objects to take on a richer and more formal structure. New GUI elements could be added to support these new canvases by extending the existing javacc grammar.

Although BioLegato is designed with biology in mind, it could be used to build software for any type of data that needs many different programs to do different tasks. Because BioLegato can run any task, from the simplest script to the most complex data pipeline, it should simplify the development of GUI applications in almost any field.

## Availability and requirements

Project name: BioLegato.

Project home page: https://github.com/bfristensky/BioLegato/wiki.

Operating systems: Linux, macOS ( > = 10.15).

Programming language: Java 8.0 or greater.

Other requirements: none.

License: Creative Commons CC-BY-NC 4.0.

Any restrictions to use by non-academics: none.

BioLegato is freely distributed as part of the BIRCH system, at http://home.cc.umanitoba.ca/%7Epsgendb/. For a quick demonstration of how BioLegato is used for common genomics tasks, see the BIRCH YouTube Channel at https://www.youtube.com/channel/UC9_3TfH3sjE0YdToVMChq-w?view_as=public. One can also download the BIRCH system using the automated install wizard and immediately try out any of the 14 BioLegato applications cited, which is best done by working through the web tutorials. For developers, a tutorial introduction to the use of PCD for programming BioLegato is found at http://home.cc.umanitoba.ca/~psgendb/birchhomedir/public_html/tutorials/bioLegato/blmenus/blmenus.html. The tutorial takes the user step by step through the process of adding a new program to an existing BioLegato application.

## Data Availability

Not applicable.

## Abbreviations

API: Application Programming Interface
GUI: Graphic User Interface
OO: Object-Oriented
PCD: stands for “Pythonesque Command Description” [8], because PCD borrows stylistically from Python
TSV: Tab-separated value file
